# Supplementary material for: The nuclear pore primes recombination-dependent DNA synthesis at arrested forks by promoting SUMO removal
Source: Nat Commun. 2020 Nov 6;11:5643. doi: 10.1038/s41467-020-19516-z (PMC7648084; doi:10.1038/s41467-020-19516-z)
Supplement: Supplementary file 1 — Supplementary Information [file 41467_2020_19516_MOESM1_ESM.pdf]

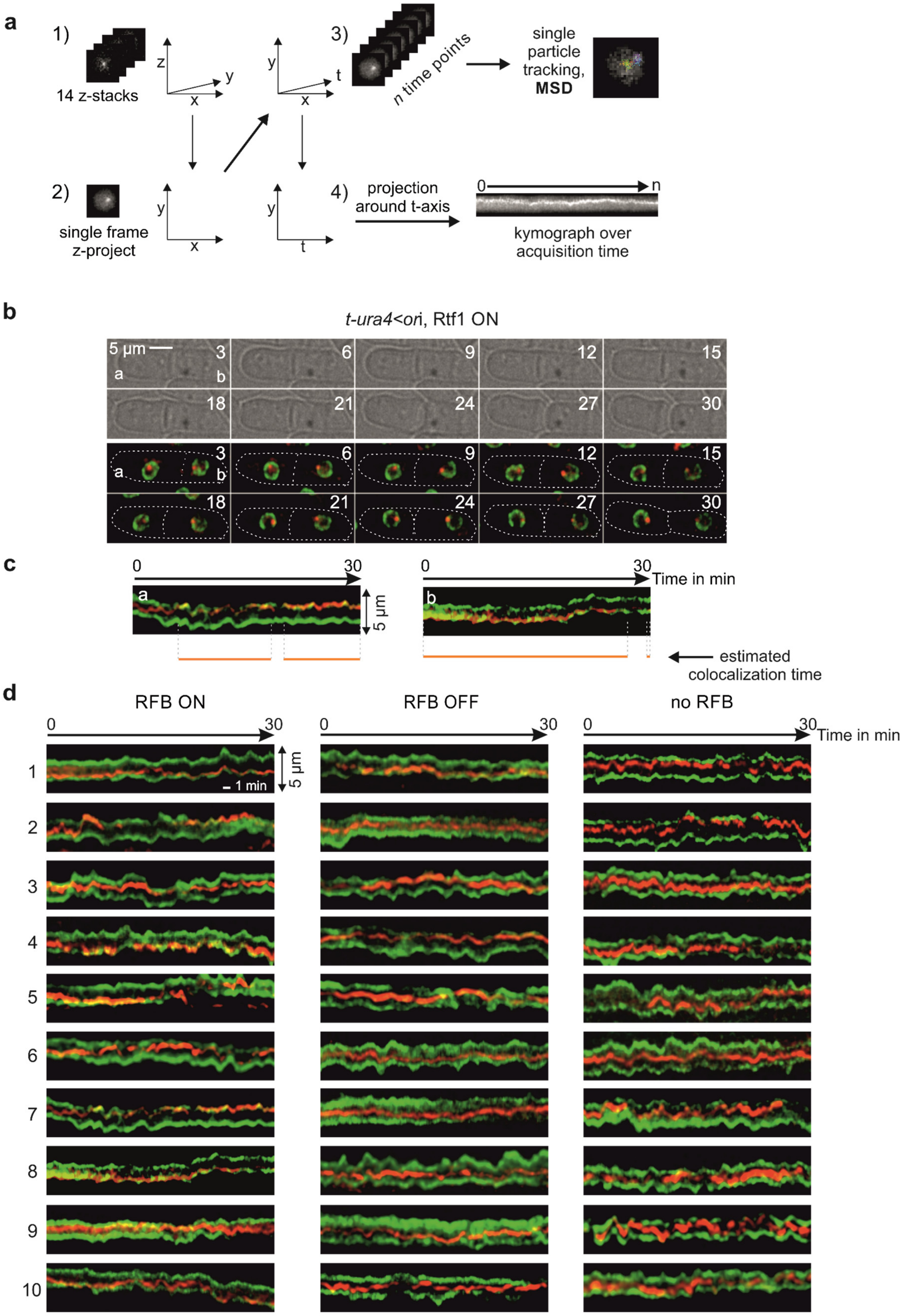

a
1)
14 z-stacks
z
y
x
2)
single frame
z-project
y
x
y
t
x
3)
n time points
single
particle
tracking,
MSD
y
t
4)
projection
around t-axis
0
n
kymograph over
acquisition time
b
t-ura4<ori, Rtf1 ON
5 µm
a
b
3
6
9
12
15
18
21
24
27
30
c
0
30
Time in min
a
b
5 µm
estimated
colocalization time
d
RFB ON
RFB OFF
no RFB
1 min
1
2
3
4
5
6
7
8
9
10

Supplementary Figure 1: Acquisition and analysis of kymographs over time (related to Fig. 1).

a Scheme of time-lapse movies acquisition and processing. (1) 14-optical slices collected over chosen time frame. (2) z-projection at each time interval. (3) 2D movie of n frames which can be subjected to single particle tracking. (4) projection over time-axis movie from point (3) generation of kymograph over acquisition time.

b Example of a 30 min processed 2D movie mounted to investigate co-localization between the *LacO*-marked RFB (red) and the nuclear periphery (Npp106-GFP).

c Example of co-localization time estimation based on kymographs constructed from movies obtained from b. Briefly, every 10 s 3D-image stacks were collected for GFP and mCherry channels. After generating a projection around the z-axis, subsequently all frames of the resulting 2D-movie were projected horizontally over the time-axis to build a kymograph for each fluorescent channel (see panel

d Kymographs used to assign co-localization time in indicated conditions. Numbers correspond cell # presented on Fig. 1f.

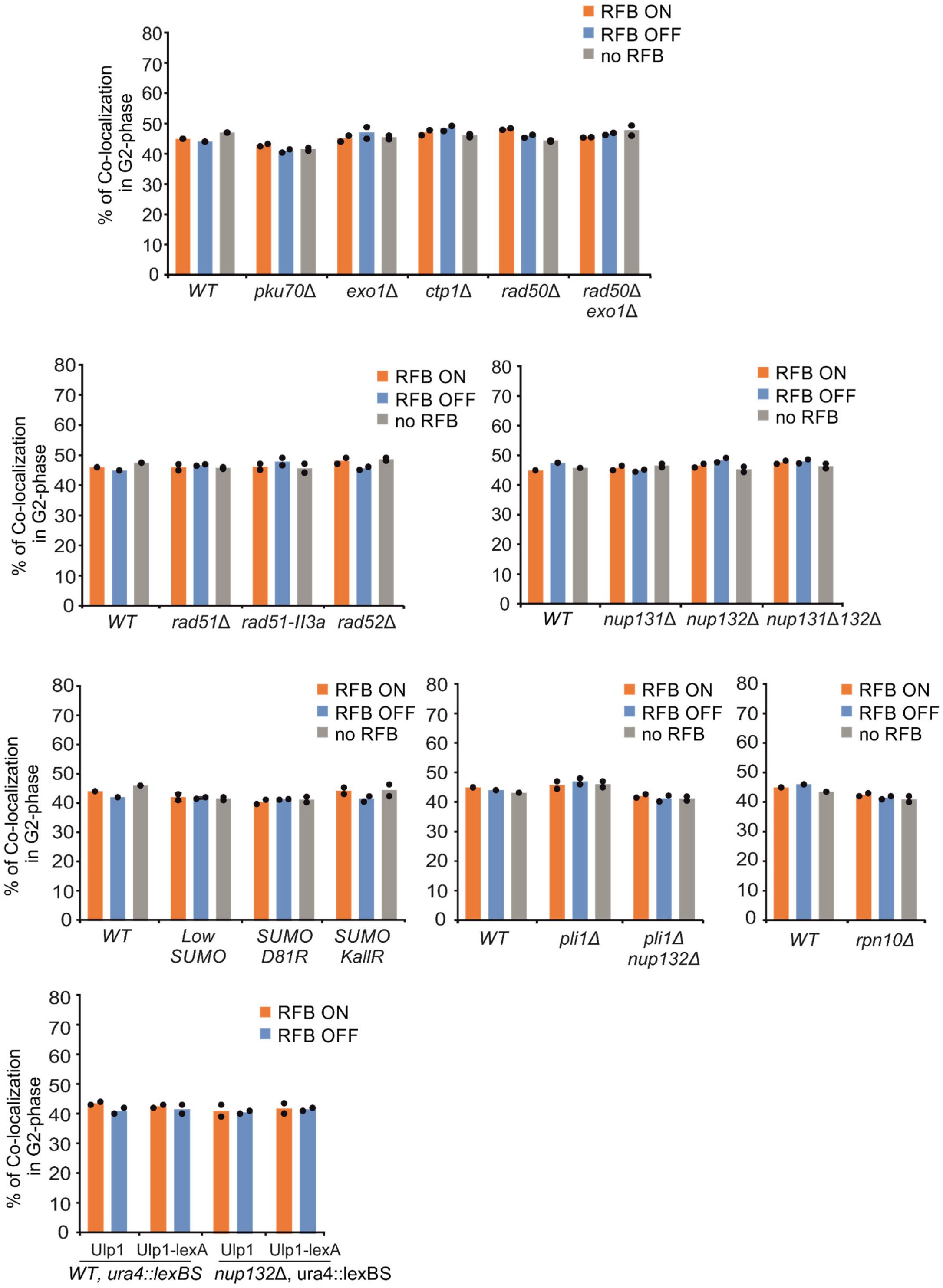

Supplementary Figure 2: Analysis of RFB relocation in G2 cells (related to Fig. 2, 4, 5 and 6).

Co-localization events in G2-phase in indicated conditions and strains, as described on Fig. 1 b-c. Two-hundred cells were analyzed for each strain and condition.

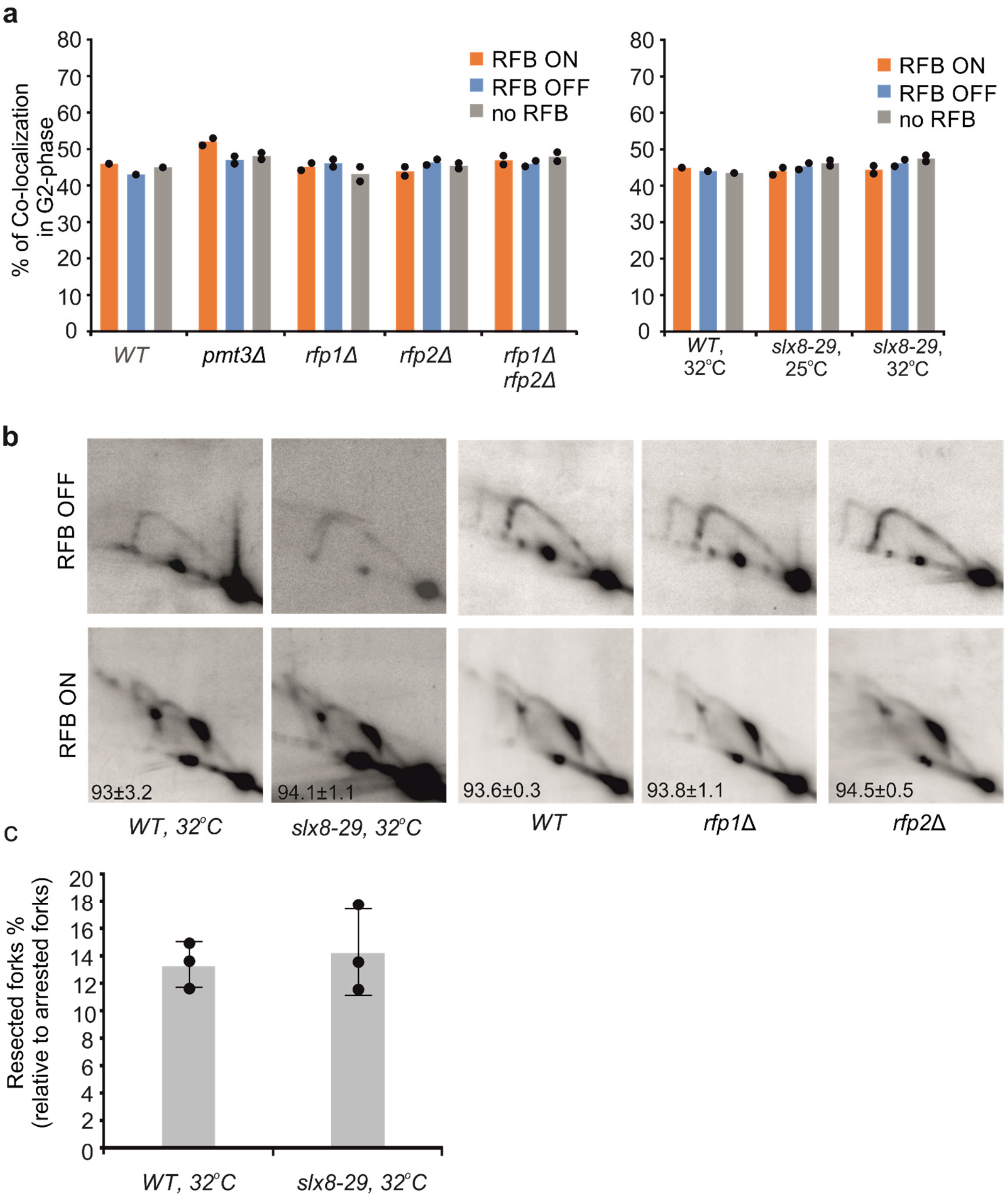

Supplementary Figure 3: Requirement of SUMO and STUBL in RDR and RFB relocation (related to Fig. 3).

a Co-localization events in in G2-phase cells in indicated conditions and strains. The *slx8-29* strains were grown at permissive (25°C) and restrictive (32°C) temperature. Dots represent values obtained from two independent biological experiments. For each set of data, *WT* strain was analyzed alongside. Two-hundred cells were analyzed for each strain and condition.

b Representative RI analysis in indicated strains and conditions as described on Fig. 3. The *ura4* gene was used as probe. Numbers indicate the % of forks blocked by the RFB ± SD.

c Quantification of resected forks. Values are means of 3 independent biological replicates ± SD.

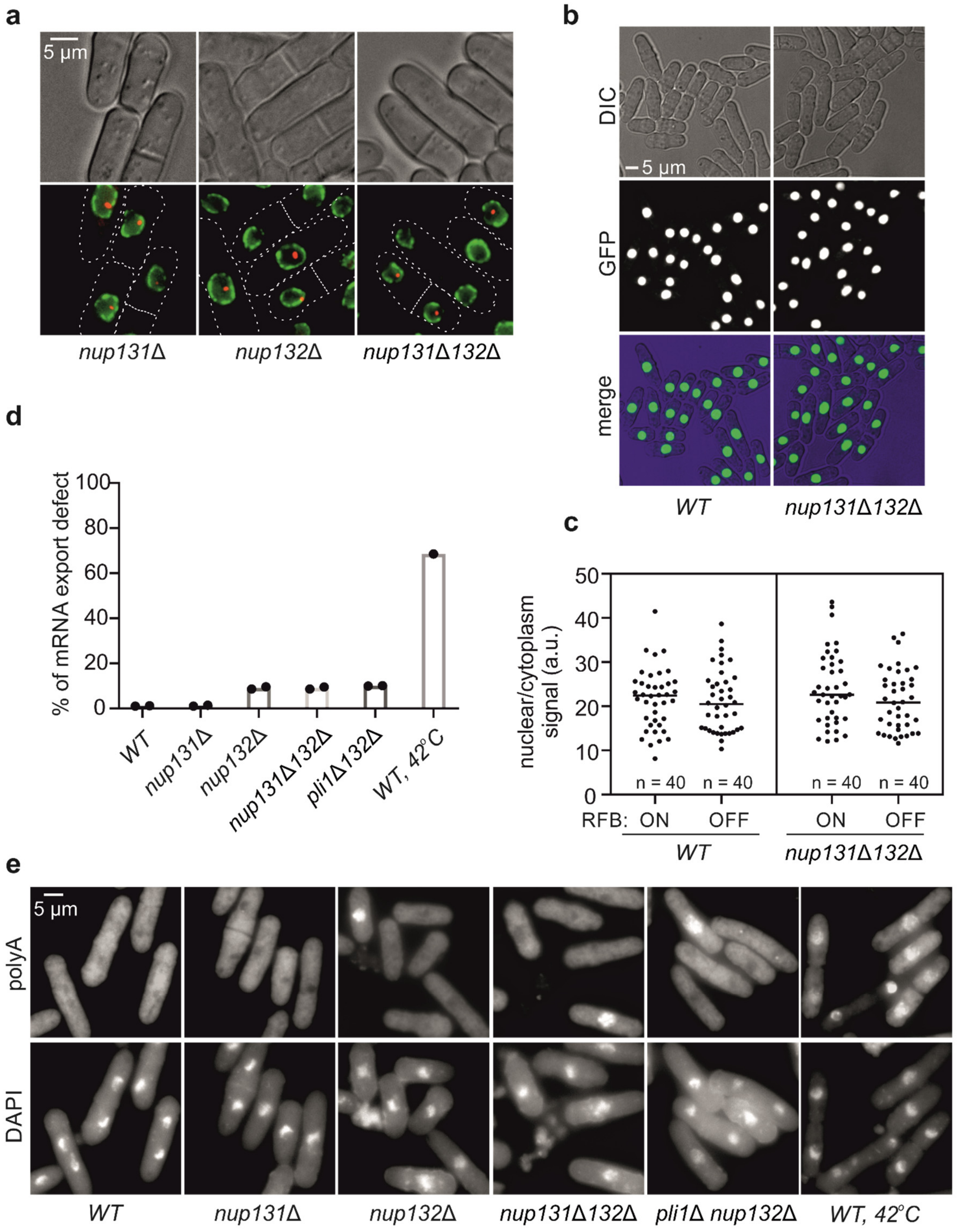

Supplementary Figure 4: Macromolecular transport in the absence of Nup132 and Nup131 (related to Fig. 4).

a Representative images of the nuclear periphery stained by Npp106-GFP (Green) and of the *LacO*-marked RFB (red) in indicated strains.
b Protein import-export was examined in the strains expressing stably LacI-NLS-GFP without *LacO* arrays integrated into the genome. Representative images obtained for *WT* and *nup131Δ nup132Δ* are shown.
c Quantification of panel b. Fluorescence intensity was measured in the nucleus and cytoplasm using ImageJ on constant volume squares to calculate ratio nuclear/cytoplasmic (N/C) signal. Each dot represents single cell ratio N/C. Bars show median N/C ratio for each group of 40 cells (RFB OFF, RFB ON).
d Percentage of cells showing mRNA export defects, as scored by the nuclear accumulation of the polyA signal in the indicated strains. *WT* heat shocked at 42°C for 30 min in water-bath was used as a positive control for mRNA export defect in one experiment. At least 200 cells were analyzed for each strain and condition. Dots correspond to values obtained from two independent biological replicates.
e Representative images, used for panel d, of subcellular localization of poly(A)+RNA examined by FISH with Cy3-oligo-dT_{50} probe in indicated strains. DAPI staining shows the position of nucleus within each cell.

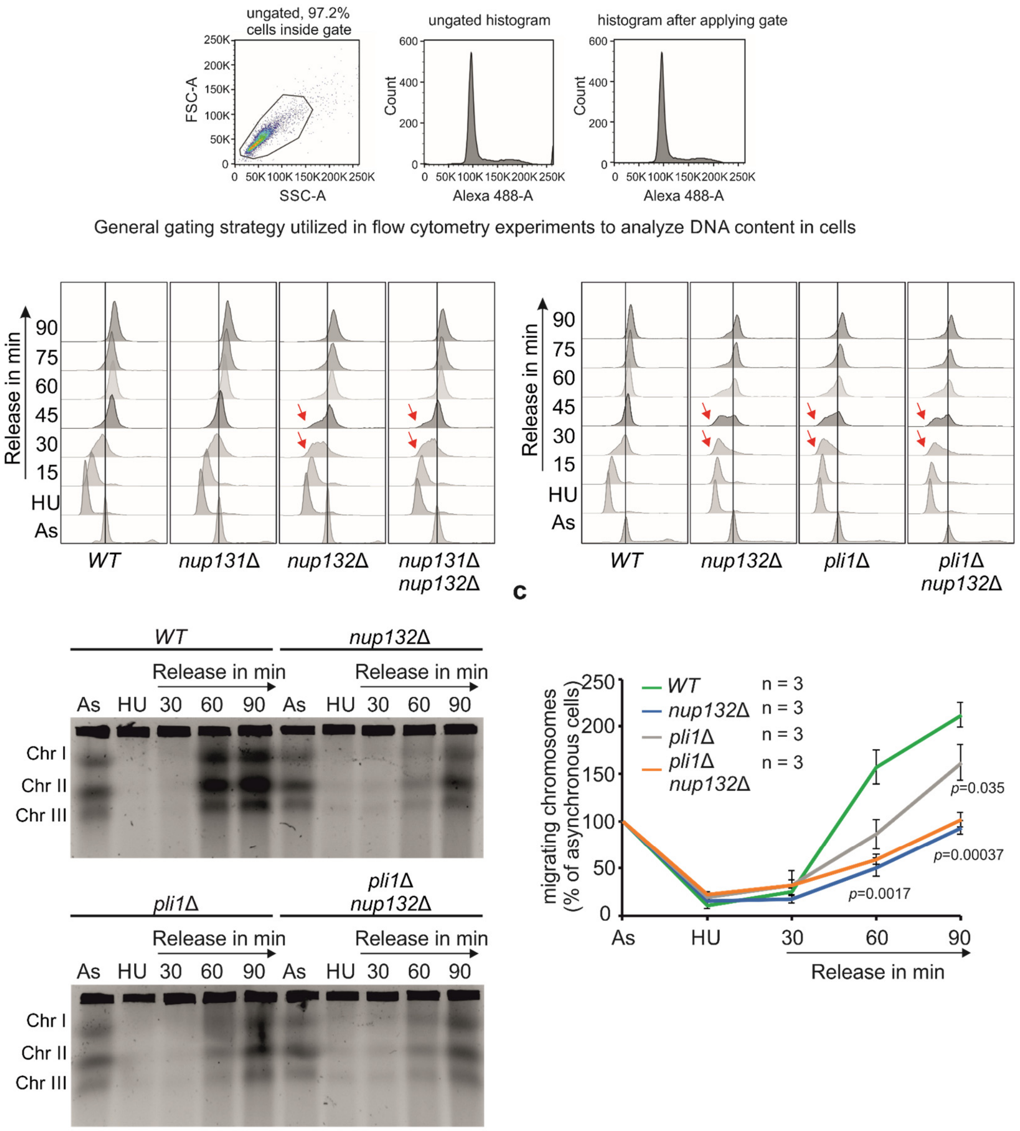

Supplementary Figure 5: Nup132 and Pli1 promote recovery from HU-stalled forks (related to Fig. 4, 5 and 7).

a Flow cytometry analysis of indicated strains in indicated conditions. Logarithmically growing cells were exposed to 20 mM HU for 4 hours and then released into fresh, HU-free, rich medium YES at 30°C to monitor S-phase progression at indicated time after release.

b Analysis of chromosome by pulse field gel electrophoresis (PFGE) in the above mentioned conditions. Representative images of chromosomes migration during PFGE in indicated strains and conditions.

c Quantification of % of chromosomes migrating into the gel after release from HU block. *p* value was calculated by two-sided t-test.

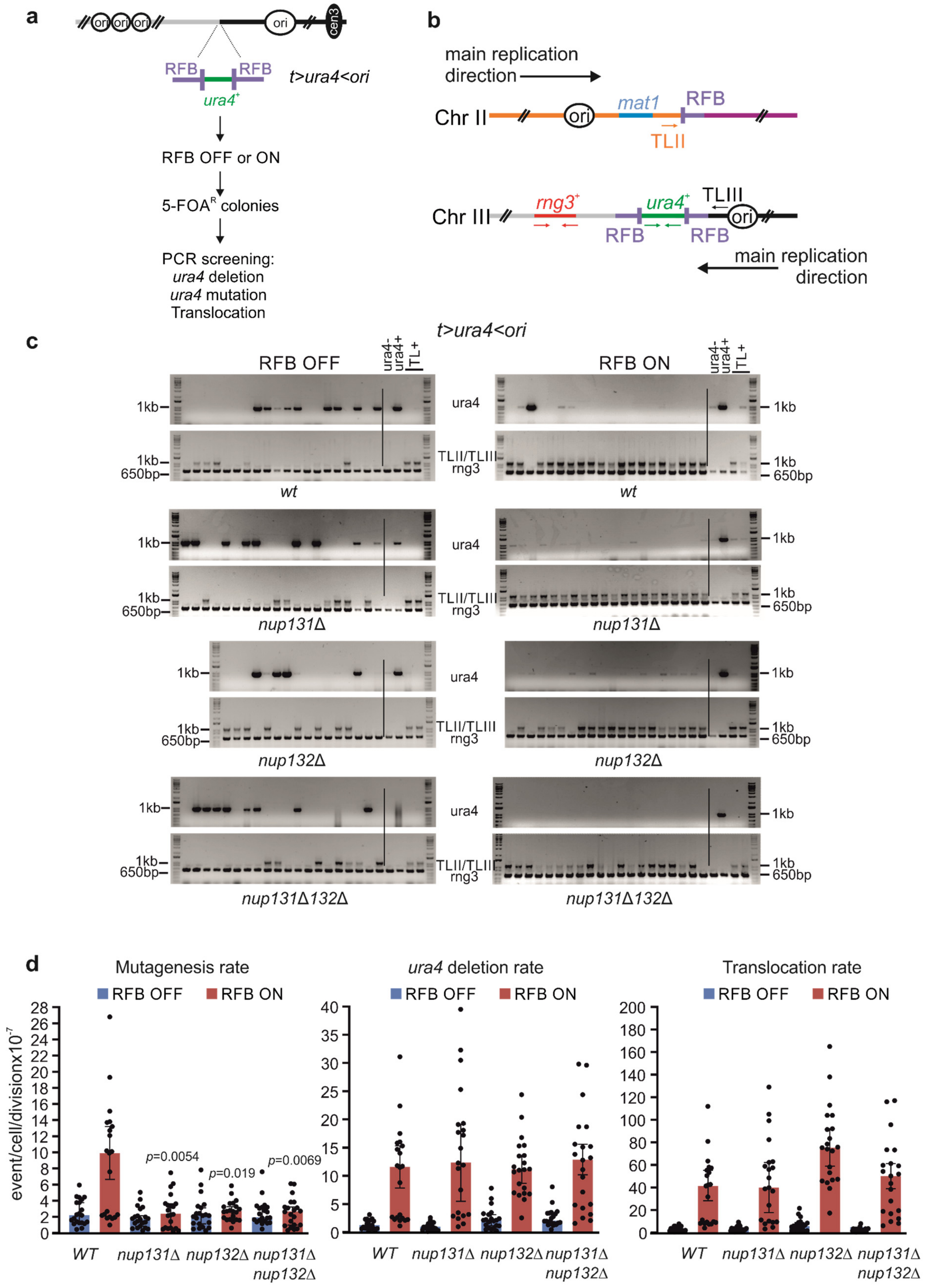
a
ori ori ori
ori
cen3
RFB RFB
ura4+
t>ura4<ori
RFB OFF or ON
5-FOA[R] colonies
PCR screening:
ura4 deletion
ura4 mutation
Translocation
b
main replication direction
Chr II
ori
mat1
RFB
TLII
Chr III
rng3+
ura4+
TLIII
ori
RFB
RFB
main replication direction
c
t>ura4<ori
RFB OFF
RFB ON
ura4-
ura4+
TL+
1kb
650bp
ura4
TLII/TLIII
rng3
wt
nup131Δ
nup132Δ
nup131Δ132Δ
d
Mutagenesis rate
ura4 deletion rate
Translocation rate
RFB OFF
RFB ON
event/cell/divisionx10-7
p=0.0054
p=0.019
p=0.0069
WT
nup131Δ
nup132Δ
nup131Δ nup132Δ

Supplementary Figure 6: Role of Nup131 and Nup132 in RFB-induced genome instability (related to Fig 4).

a Diagram of the *t>ura4<ori* construct contacting two *RTS1*-RFB (purple bars) in inverted repeat orientation at both side of the *ura4⁺* gene (in green). After several generations upon fork arrest (RFB ON) or not (RFB OFF), the genetic loss of *ura4⁺* was selected on 5FOA-containing media. Independent $5FOA^R$ colonies were analyzed by PCR to identify *ura4* deletion, *ura4* mutation and translocation.

b Diagram of the *t>ura4<ori* construct containing two *RTS1*-RFB (purple bars) in inverted repeat orientation at both sides of the *ura4⁺* gene (in green) on chromosome III and of the chromosome II harboring the *RTS1* sequence at its natural location, near the *mat1* locus. Primers used for amplifying 1 kb *ura4* fragment and the 650 bp *rng3* fragment are represented in green and red, respectively. Primers used for amplifying translocation junction (1.2 kb) are depicted in orange on chromosome II (TL II) and black on chromosome III (TL III).

c Example of PCR-based screening to detect deletion, mutation and translocation events in indicated strains and conditions. The *rng3* PCR product was used as a control of positive PCR amplification together with the TLII-TLIII primers to detect translocation junctions. Strains harboring a deletion of the *ura4* gene (*ura4-D18*), or wild type *ura4⁺*, or stable translocations were used as controls of PCR amplification.

d Rate of mutagenesis (left panel), deletion (middle) and translocation (right) in indicated strains and conditions. Each dot correspond to one value from 21 independent cultures. Values are median ± 95 % confidence interval. *p* value was calculated using the two-sided Mann & Whitney U test.

Table S1: Strains used in this study

Strain number	Mating type	Genotype	Reference
**SL1524**	*h-*	*npp106-GFP:Nat arg3::mCherry-LacI nmt41:rtf1:sup35 ade6-704 leu1-32 t-LacO 7,9Kb:Kan:ura4*$^+$*<ori (uraR)*	this study
**KK47**	*h-*	*npp106-GFP:Nat arg3::mCherry-LacI nmt41:rtf1:sup35 ade6-704 leu1-32 t-LacO 7,9Kb:Kan:ura4*$^+$*-ori (ura4+, no RTS1)*	this study
**AS90**	*h-*	*npp106-GFP:Nat arg3::psv40:GFP-LacI nmt41:rtf1:sup35 ade6-704 leu1-32 LacO7.9Kb:Kan:ura4+<ori*	this study
**KK434**	*h-*	*npp106-GFP:Nat nmt41:rtf1:sup35 ade6-704 leu1-32 t-ura4-SD20<ori (uraR)*	this study
**KK327**	*h+*	*man1-GFP:Nat nmt41:rtf1:sup35 ade6-704 leu1-32 t-ura4-SD20<ori (uraR)*	this study
**KK481**	*h-*	*sad1-GFP nmt41:rtf1:sup35 ade6-704 leu1-32 t-ura4-SD20<ori (uraR)*	this study
**KK1296**	*h-*	*pku70::Hygro npp106-GFP:Nat arg3::mCherry-LacI nmt41:rtf1:sup35 ade6-704 leu1-32 t-LacO 7,9Kb:Kan:ura4*$^+$*<ori (uraR)*	this study
**KK1313**	*h+*	*pku70::Hygro npp106-GFP:Nat arg3::mCherry-LacI nmt41:rtf1:sup35 ade6-704 leu1-32 t-LacO 7,9Kb:Kan:ura4*$^+$*-ori (ura4, no RTS1)*	this study
**KK43**	*h+*	*exo1::Nat npp106-GFP:Nat arg3::mCherry-LacI nmt41:rtf1:sup35 ade6-704 leu1-32 t-LacO 7,9Kb:Kan:ura4*$^+$*<ori (uraR)*	this study
**KK85**	*h-*	*exo1::Nat npp106-GFP:Nat arg3::mCherry-LacI nmt41:rtf1:sup35 ade6-704 leu1-32 t-LacO 7,9Kb:Kan:ura4*$^+$*-ori (ura4, no RTS1)*	this study
**KK507**	*h+*	*ctp1::Hygro npp106-GFP:Nat arg3::mCherry-LacI nmt41:rtf1:sup35 ade6-704 leu1-32 t-LacO 7,9Kb:Kan:ura4*$^+$*<ori (uraR)*	this study
**KK509**	*h+*	*ctp1::Hygro npp106-GFP:Nat arg3::mCherry-LacI nmt41:rtf1:sup35 ade6-704 leu1-32 t-LacO 7,9Kb:Kan:ura4*$^+$*-ori (ura4, no RTS1)*	this study
**KK143**	*h-*	*rad50::Kan npp106-GFP:Nat arg3::mCherry-LacI nmt41:rtf1:sup35 ade6-704 leu1-32 t-LacO 7,9Kb:Kan:ura4*$^+$*<ori (uraR)*	this study
**KK144**	*h+*	*rad50::Kan npp106-GFP:Nat arg3::mCherry-LacI nmt41:rtf1:sup35 ade6-704 leu1-32 t-LacO 7,9Kb:Kan:ura4*$^+$*-ori (ura4, no RTS1)*	this study
**KK147**	*h-*	*exo1::Nat rad50::Kan npp106-GFP:Nat arg3::mCherry-LacI nmt41:rtf1:sup35 ade6-704 leu1-32 t-LacO 7,9Kb:Kan:ura4*$^+$*<ori (uraR)*	this study
**KK150**	*h+*	*exo1::Nat rad50::Kan npp106-GFP:Nat arg3::mCherry-LacI nmt41:rtf1:sup35 ade6-704 leu1-32 t-LacO 7,9Kb:Kan:ura4*$^+$*-ori (ura4, no RTS1)*	this study
**KK170**	*h+*	*rad51::Kan npp106-GFP:Nat arg3::mCherry-LacI nmt41:rtf1:sup35 ade6-704 leu1-32 t-LacO 7,9Kb:Kan:ura4*$^+$*<ori (uraR)*	this study
**KK172**	*h-*	*rad51::Kan npp106-GFP:Nat arg3::mCherry-LacI nmt41:rtf1:sup35 ade6-704 leu1-32 t-LacO 7,9Kb:Kan:ura4*$^+$*-ori (ura4, no RTS1)*	this study
**KK526**	*h-*	*loxP:rad51 R152A-R324A- K334A:LoxM3 (rad51-3A) npp106-GFP:Nat arg3::mCherry-LacI nmt41:rtf1:sup35 ade6-704 leu1-32 t-LacO 7,9Kb:Kan:ura4*$^+$*<ori (uraR)*	this study
**KK529**	*h+*	*loxP:rad51 R152A-R324A- K334A:LoxM3 (rad51-3A) npp106-GFP:Nat arg3::mCherry-LacI nmt41:rtf1:sup35 ade6-704 leu1-32 t-LacO 7,9Kb:Kan:ura4*$^+$*-ori (ura4, no RTS1)*	this study

**KK59**	h-	rad52::Kan npp106-GFP:Nat arg3::mCherry-LacI nmt41:rtf1:sup35 ade6-704 leu1-32 t-LacO 7,9Kb:Kan:ura4^{+}<ori (uraR)	this study
**KK58**	h-	rad52::Kan npp106-GFP:Nat arg3::mCherry-LacI nmt41:rtf1:sup35 ade6-704 leu1-32 t-LacO 7,9Kb:Kan:ura4^{+}-ori (ura4, no RTS1)	this study
**KK1281**	h-	pku70::Hygro npp106-GFP:Nat nmt41:rtf1:sup35 ade6-704 leu1-32 t-ura4-SD20<ori (uraR)	this study
**KK1292**	h-	exo1::Nat npp106-GFP:Nat nmt41:rtf1:sup35 ade6-704 leu1-32 t-ura4-SD20<ori (uraR)	this study
**KK1278**	h+	rad50::Kan npp106-GFP:Nat nmt41:rtf1:sup35 ade6-704 leu1-32 t-ura4-SD20<ori (uraR)	this study
**KK1287**	h-	loxP:rad51 R152A-R324A- K334A:LoxM3 (rad51-3A) npp106-GFP:Nat nmt41:rtf1:sup35 ade6-704 leu1-32 t-ura4-SD20<ori (uraR)	this study
**YC6**	h-	t-ura4-SD20-ori nmt41:rtf1:sup35 ade6-704 leu1-32	7
**YC13**	h-	t-ura4-SD20<ori nmt41:rtf1:sup35 ade6-704 leu1-32	7
**SL1409**	h-	slx8-29:Hygro npp106-GFP:Nat arg3::mCherry-lacI rtf1:nmt41:sup35 t-lacO7,9kb:kan-ura4+<ori ade6-704 leu1-32	this study
**KK92**	h+	slx8-29-Hygro npp106-GFP:Nat arg3::mCherry-LacI nmt41:rtf1:sup35 ade6-704 leu1-32 t-LacO 7,9Kb:Kan:ura4^{+-}ori (ura+, no RTS1)	this study
**KK105**	h+	pmt3::Hygro npp106-GFP:Nat arg3::mCherry-LacI nmt41:rtf1:sup35 ade6-704 leu1-32 t-LacO 7,9Kb:Kan:ura4^{+}<ori (uraR)	this study
**KK464**	h-	npp106-GFP:Nat arg3::mCherry-LacI nmt41:rtf1:sup35 ade6-704 leu1-32 his3D1 t-LacO 7,9Kb:Kan:ura4^{+}<ori (uraR)	this study
**KK460**	h+	rfp1::his3 npp106-GFP:Nat arg3::mCherry-LacI nmt41:rtf1:sup35 ade6-704 leu1-32 his3D1 t-LacO 7,9Kb:Kan:ura4^{+}<ori (uraR)	this study
**KK463**	h+	rfp2::Hygro npp106-GFP:Nat arg3::mCherry-LacI nmt41:rtf1:sup35 ade6-704 leu1-32 his3D1 t-LacO 7,9Kb:Kan:ura4^{+}<ori (uraR)	this study
**KK465**	h-	rfp1::his3 rfp2::Hygro npp106-GFP:Nat arg3::mCherry-LacI nmt41:rtf1:sup35 ade6-704 leu1-32 his3D1 t-LacO 7,9Kb:Kan:ura4^{+}<ori (uraR)	this study
**KK662**	h+	npp106-GFP:Nat arg3::mCherry-LacI nmt41:rtf1:sup35 ade6-704 leu1-32 his3D1 t-LacO 7,9Kb:Kan:ura4^{+}-ori (ura4, no RTS1)	this study
**KK663**	h-	rfp1::his3 npp106-GFP:Nat arg3::mCherry-LacI nmt41:rtf1:sup35 ade6-704 leu1-32 his3D1 t-LacO 7,9Kb:Kan:ura4^{+}-ori (ura4, no RTS1)	this study
**KK164**	h-	rfp2::Hygro npp106-GFP:Nat arg3::mCherry-LacI nmt41:rtf1:sup35 ade6-704 leu1-32 t-LacO 7,9Kb:Kan:ura4^{+}-ori (ura4, no RTS1)	this study
**KK661**	h+	rfp1::his3 rfp2::Hygro npp106-GFP:Nat arg3::mCherry-LacI nmt41:rtf1:sup35 ade6-704 leu1-32 his3D1 t-LacO 7,9Kb:Kan:ura4^{+}-ori (ura4, no RTS1)	this study
**KK1018**	h+	slx8-29-Hygro npp106-GFP:Nat arg3::psv40:GFP-LacI nmt41:rtf1:sup35 ade6-704 leu1-32 LacO7.9Kb:Kan:ura4+<ori	this study
**YC297**	h+	slx8-29:Hygro nmt41:rtf1:sup35 ade6-704 leu1-32 t-ura4-SD20-ori (ura4+, no RTS1)	this study
**YC301**	h+	slx8-29:Hygro nmt41:rtf1:sup35 ade6-704 leu1-32 t-ura4-SD20<ori (uraR)	this study
**KK110**	h+	nup131::Hygro npp106-GFP:Nat arg3::mCherry-LacI nmt41:rtf1:sup35 ade6-704 leu1-32 t-LacO 7,9Kb:Kan:ura4^{+}<ori (uraR)	this study

**KK112**	*h-*	*nup131::Hygro npp106-GFP:Nat arg3::mCherry-LacI nmt41:rtf1:sup35 ade6-704 leu1-32 t-LacO 7,9Kb:Kan:ura4^{+}-ori (ura4, no RTS1)*	this study
**KK114**	*h-*	*nup132::Hygro npp106-GFP:Nat arg3::mCherry-LacI nmt41:rtf1:sup35 ade6-704 leu1-32 t-LacO 7,9Kb:Kan:ura4^{+}<ori (uraR)*	this study
**KK116**	*h+*	*nup132::Hygro npp106-GFP:Nat arg3::mCherry-LacI nmt41:rtf1:sup35 ade6-704 leu1-32 t-LacO 7,9Kb:Kan:ura4^{+}-ori (ura4, no RTS1)*	this study
**KK118**	*h+*	*nup131::Hygro nup132::Hygro npp106-GFP:Nat arg3::mCherry-LacI nmt41:rtf1:sup35 ade6-704 leu1-32 t-LacO 7,9Kb:Kan:ura4^{+}<ori (uraR)*	this study
**KK119**	*h-*	*nup131::Hygro nup132::Hygro npp106-GFP:Nat arg3::mCherry-LacI nmt41:rtf1:sup35 ade6-704 leu1-32 t-LacO 7,9Kb:Kan:ura4^{+}-ori (ura4, no RTS1)*	this study
**KK261**	*h+*	*nup131::Hygro nmt41:rtf1:sup35 ade6-704 leu1-32 t-ura4^{+}-ori (ura4, no RTS1)*	this study
**KK266**	*h-*	*nup132::Nat nmt41:rtf1:sup35 ade6-704 leu1-32 t-ura4^{+}-ori (ura4, no RTS1)*	this study
**KK271**	*h-*	*nup131::Hygro nup132::Nat nmt41:rtf1:sup35 ade6-704 leu1-32 t-ura4^{+}-ori (ura4, no RTS1)*	this study
**KK222**	*h-*	*nup131::Hygro nmt41:rtf1:sup35 ade6-704 leu1-32 t-ura4^{+}<ori (uraR)*	this study
**KK227**	*h+*	*nup132::Nat nmt41:rtf1:sup35 ade6-704 leu1-32 t-ura4^{+}<ori (uraR)*	this study
**KK232**	*h+*	*nup131::Hygro nup132::Nat nmt41:rtf1:sup35 ade6-704 leu1-32 t-ura4^{+}<ori (uraR)*	this study
**KK1254**	*h+*	*nup132::Nat npp106-GFP:Nat nmt41:rtf1:sup35 ade6-704 leu1-32 t-ura4-SD20<ori (uraR)*	this study
**KK440**	*h-*	*nup131::Hygro nup132::Nat npp106-GFP:Nat nmt41:rtf1:sup35 ade6-704 leu1-32 t-ura4-SD20<ori (uraR)*	this study
**KK531**	*h-*	*nmt41:rtf1:sup35 ade6-704 leu1-32 t>ura4^{+}<ori (RuraR)*	this study
**KK535**	*h-*	*nup131::Hygro nmt41:rtf1:sup35 ade6-704 leu1-32 t>ura4^{+}<ori (RuraR)*	this study
**KK539**	*h-*	*nup132::Nat nmt41:rtf1:sup35 ade6-704 leu1-32 t>ura4^{+}<ori (RuraR)*	this study
**KK543**	*h-*	*nup131::Hygro nup132::Nat nmt41:rtf1:sup35 ade6-704 leu1-32 t>ura4^{+}<ori (RuraR)*	this study
**KK810**	*h+*	*arg3::psv40:GFP-LacI nmt41:rtf1:sup35 ade6-704 leu1-32 t-ura4-SD20<ori (uraR)*	this study
**KK814**	*h+*	*nup131::Hygro nup132::Hygro arg3::psv40:GFP-LacI nmt41:rtf1:sup35 ade6-704 leu1-32 t-ura4-SD20<ori (uraR)*	this study
**KK936**	*h+*	*pli1::Hygro nmt41:rtf1:sup35 ade6-704 leu1-32 t-ura4-SD20<ori (uraR)*	this study
**KK957**	*h-*	*pli1::Hygro nmt41:rtf1:sup35 ade6-704 leu1-32 t-ura4-SD20-ori (ura4, no RTS1)*	this study
**KK940**	*h-*	*pli1::Hygro nup132::Nat nmt41:rtf1:sup35 ade6-704 leu1-32 t-ura4-SD20<ori (uraR)*	this study
**KK965**	*h+*	*pli1::Hygro nup132::Nat nmt41:rtf1:sup35 ade6-704 leu1-32 t-ura4-SD20-ori (ura4, no RTS1)*	this study
**KK154**	*h-*	*pli1::Hygro npp106-GFP:Nat arg3::mCherry-LacI nmt41:rtf1:sup35 ade6-704 leu1-32 t-LacO 7,9Kb:Kan:ura4^{+}<ori (uraR)*	this study
**KK156**	*h+*	*pli1::Hygro npp106-GFP:Nat arg3::mCherry-LacI nmt41:rtf1:sup35 ade6-704 leu1-32 t-LacO 7,9Kb:Kan:ura4^{+}-ori (ura4, no RTS1)*	this study
**KK1086**	*h+*	*pli1::Hygro nup132::Hygro npp106-GFP:Nat arg3::mCherry-LacI nmt41:rtf1:sup35 ade6-704 leu1-32 t-LacO 7,9Kb:Kan:ura4^{+}<ori (uraR)*	this study

**KK1176**	*h-*	*pli1::Hygro nup132::Hygro npp106-GFP:Nat arg3::mCherry-LacI nmt41:rtf1:sup35 ade6-704 leu1-32 t-LacO 7,9Kb:Kan:ura4*$^{+}$*-ori (ura4, no RTS1)*	this study
**KK1100**	*h-*	*pli1::Hygro npp106-GFP:Nat arg3::psv40:GFP-LacI nmt41:rtf1:sup35 ade6-704 leu1-32 t-LacO 7,9Kb:Kan:ura4*$^{+}$*<ori (uraR)*	this study
**KK1256**	*h-*	*pli1::Hygro npp106-GFP:Nat nmt41:rtf1:sup35 ade6-704 leu1-32 t-ura4-SD20<ori (uraR)*	this study
**KK1258**	*h+*	*pli1::Hygro nup132::Nat npp106-GFP:Nat nmt41:rtf1:sup35 ade6-704 leu1-32 t-ura4-SD20<ori (uraR)*	this study
**KK1029**	*h-*	*pli1::Hygro slx8-29:Hygro nmt41:rtf1:sup35 ade6-704 leu1-32 t-ura4*$^{+}$*<ori (uraR)*	this study
**KK1032**	*h+*	*pli1::Hygro slx8-29:Hygro nmt41:rtf1:sup35 ade6-704 leu1-32 t-ura4-SD20-ori (ura4, no RTS1)*	this study
**KK1157**	*h-*	*Low SUMO (tetO7-TATACYC1 pmt3:Kan) npp106-GFP:Nat arg3::mCherry-LacI nmt41:rtf1:sup35 ade6-704 leu1-32 t-LacO 7,9Kb:Kan:ura4*$^{+}$*<ori (uraR)*	this study
**KK1159**	*h-*	*Low SUMO (tetO7-TATACYC1 pmt3:Kan) npp106-GFP:Nat arg3::mCherry-LacI nmt41:rtf1:sup35 ade6-704 leu1-32 t-LacO 7,9Kb:Kan:ura4*$^{+}$*-ori (ura4, no RTS1)*	this study
**KK1161**	*h+*	*SUMO-D81R (pmt3D81R) npp106-GFP:Nat arg3::mCherry-LacI nmt41:rtf1:sup35 ade6-704 leu1-32 t-LacO 7,9Kb:Kan:ura4*$^{+}$*<ori (uraR)*	this study
**KK1164**	*h+*	*SUMO-D81R (pmt3D81R) npp106-GFP:Nat arg3::mCherry-LacI nmt41:rtf1:sup35 ade6-704 leu1-32 t-LacO 7,9Kb:Kan:ura4*$^{+}$*-ori (ura4, no RTS1)*	this study
**KK1051**	*h-*	*Low SUMO (tetO7-TATACYC1 pmt3:Kan) nmt41:rtf1:sup35 ade6-704 leu1-32 t-ura4-SD20<ori (uraR)*	this study
**KK1046**	*h-*	*Low SUMO (tetO7-TATACYC1 pmt3:Kan) nmt41:rtf1:sup35 ade6-704 leu1-32 t-ura4-SD20-ori (ura4, noRTS1)*	this study
**KK1052**	*h-*	*Low SUMO (tetO7-TATACYC1 pmt3:Kan) nup132::Nat nmt41:rtf1:sup35 ade6-704 leu1-32 t-ura4-SD20<ori (uraR)*	this study
**KK1045**	*h-*	*Low SUMO (tetO7-TATACYC1 pmt3:Kan) nup132::Nat nmt41:rtf1:sup35 ade6-704 leu1-32 t-ura4-SD20-ori (ura4, noRTS1)*	this study
**KK1067**	*h+*	*SUMO-D81R (pmt3D81R) nmt41:rtf1:sup35 ade6-704 leu1-32 t-ura4-SD20<ori (uraR)*	this study
**KK1069**	*h+*	*SUMO-D81R (pmt3D81R) nup132::Nat nmt41:rtf1:sup35 ade6-704 leu1-32 t-ura4-SD20<ori (uraR)*	this study
**KK1075**	*h-*	*SUMO-D81R (pmt3D81R) nmt41:rtf1:sup35 ade6-704 leu1-32 t-ura4-SD20-ori (ura4, noRTS1)*	this study
**KK1076**	*h-*	*nup132::Nat SUMO-D81R (pmt3D81R) nmt41:rtf1:sup35 ade6-704 leu1-32 t-ura4-SD20-ori (ura4, noRTS1)*	this study
**KK1350**	*h-*	*SUMO-KallR (pmt3-KallR) npp106-GFP:Nat arg3::mCherry-LacI nmt41:rtf1:sup35 ade6-704 leu1-32 t-LacO 7,9Kb:Kan:ura4*$^{+}$*<ori (uraR)*	this study
**KK1353**	*h-*	*SUMO-KallR (pmt3-KallR) npp106-GFP:Nat arg3::mCherry-LacI nmt41:rtf1:sup35 ade6-704 leu1-32 t-LacO 7,9Kb:Kan:ura4*$^{+}$*-ori (ura4, no RTS1)*	this study
**KK1340**	*h+*	*SUMO-KallR (pmt3-KallR) nmt41:rtf1:sup35 ade6-704 leu1-32 t-ura4-SD20<ori (uraR)*	this study
**KK1351**	*h-*	*SUMO-KallR (pmt3-KallR) nmt41:rtf1:sup35 ade6-704 leu1-32 t-ura4-SD20-ori (ura4, noRTS1)*	this study
**KK1345**	*h+*	*nup132::Nat SUMO-KallR (pmt3-KallR) nmt41:rtf1:sup35 ade6-704 leu1-32 t-ura4-SD20<ori (uraR)*	this study

**KK1352**	*h-*	*nup132::Nat SUMO-KallR (pmt3-KallR) nmt41:rtf1:sup35 ade6-704 leu1-32 t-ura4-SD20-ori (ura4, noRTS1)*	this study
**KK1151**	*h+*	*rpn10::Hygro npp106-GFP:Nat arg3::mCherry-LacI nmt41:rtf1:sup35 ade6-704 leu1-32 t-LacO 7,9Kb:Kan:ura4*$^{+}$*<ori (uraR)*	this study
**KK1154**	*h-*	*rpn10::Hygro npp106-GFP:Nat arg3::mCherry-LacI nmt41:rtf1:sup35 ade6-704 leu1-32 t-LacO 7,9Kb:Kan:ura4*$^{+}$*-ori (ura4, no RTS1)*	this study
**KK1079**	*h-*	*rpn10::Hygro nmt41:rtf1:sup35 ade6-704 leu1-32 t-ura4-SD20<ori (uraR)*	this study
**KK1081**	*h+*	*rpn10::Hygro nmt41:rtf1:sup35 ade6-704 leu1-32 t-ura4-SD20-ori (ura4, noRTS1)*	this study
**KK1082**	*h+*	*rpn10::Hygro nup132::Nat nmt41:rtf1:sup35 ade6-704 leu1-32 t-ura4-SD20<ori (uraR)*	this study
**KK1085**	*h-*	*rpn10::Hygro nup132::Nat nmt41:rtf1:sup35 ade6-704 leu1-32 t-ura4-SD20-ori (ura4, noRTS1)*	this study
**KK1047**	*h-*	*pnmt81-ulp1:Kan nmt41:rtf1:sup35 ade6-704 leu1-32 t-ura4-SD20<ori (uraR)*	this study
**KK1043**	*h+*	*pnmt81-ulp1:Kan nmt41:rtf1:sup35 ade6-704 leu1-32 t-ura4-SD20-ori (ura4, no RTS1)*	this study
**KK1049**	*h-*	*pnmt81-ulp1:Kan nup132::Nat nmt41:rtf1:sup35 ade6-704 leu1-32 t-ura4-SD20<ori (uraR)*	this study
**KK1044**	*h-*	*pnmt81-ulp1:Kan nup132::Nat nmt41:rtf1:sup35 ade6-704 leu1-32 t-ura4-SD20-ori (ura4, no RTS1)*	this study
**KK1192**	*h+*	*npp106-GFP:Nat arg3::mCherry-LacI nmt41:rtf1:sup35 ade6-704 leu1-32 t-LacO 7,9Kb:Kan:ura4::LexBS<ori (uraR)*	this study
**KK1193**	*h-*	*ulp1-lexA:Hygro npp106-GFP:Nat arg3::mCherry-LacI nmt41:rtf1:sup35 ade6-704 leu1-32 t-LacO 7,9Kb:Kan:ura4::LexBS<ori (uraR)*	this study
**KK1194**	*h+*	*nup132::Hygro npp106-GFP:Nat arg3::mCherry-LacI nmt41:rtf1:sup35 ade6-704 leu1-32 t-LacO 7,9Kb:Kan:ura4::LexBS<ori (uraR)*	this study
**KK1195**	*h-*	*nup132::Hygro ulp1-lexA:Hygro npp106-GFP:Nat arg3::mCherry-LacI nmt41:rtf1:sup35 ade6-704 leu1-32 t-LacO 7,9Kb:Kan:ura4::LexBS<ori (uraR)*	this study
**KK1122**	*h-*	*nmt41:rtf1:sup35 ade6-704 leu1-32 t-Kan-lexBS:ura4-SD20<ori (uraR)*	this study
**KK1121**	*h-*	*ulp1-lexA:Hygro nmt41:rtf1:sup35 ade6-704 leu1-32 t-ura4-SD20<ori (uraR)*	this study
**KK1131**	*h-*	*ulp1-lexA:Hygro nmt41:rtf1:sup35 ade6-704 leu1-32 t-Kan-lexBS:ura4-SD20<ori (uraR)*	this study
**KK1139**	*h-*	*nup132::Nat nmt41:rtf1:sup35 ade6-704 leu1-32 t-Kan-lexBS:ura4-SD20<ori (uraR)*	this study
**KK1137**	*h-*	*nup132::Nat ulp1-lexA:Hygro nmt41:rtf1:sup35 ade6-704 leu1-32 t-ura4-SD20<ori (uraR)*	this study
**KK1141**	*h-*	*nup132::Nat ulp1-lexA :Hygro nmt41:rtf1:sup35 ade6-704 leu1-32 t-Kan-lexBS:ura4-SD20<ori (uraR)*	this study
**KK697**	*h-*	*ulp1::Kan nmt41:rtf1:sup35 ade6-704 leu1-32 t-ura4-SD20<ori (uraR)*	this study
**KK442**	*h-*	*ssb3-YFP:Nat nmt41:rtf1:sup35 ade6-704 leu1-32 t-ura4-SD20<ori (uraR)*	this study
**KK1097**	*h-*	*pli1::Hygro ssb3-YFP:Nat nmt41:rtf1:sup35 ade6-704 leu1-32 t-ura4*$^{+}$*<ori (uraR)*	this study

Table S2: primers used in this study

Name	Distance (bp) from the *RTS1*-RFB position	Sequence (5'-3')	Experiment
L5F	-110	AGGGCATTAAGGCTTATTTACAGA	ChIP (all experiments)
L5R		TCACGTTTAATTTCAAACATCCA	ChIP (all experiments)
L3F	110	TTTAAATCAAATCTTCCATGCG	ChIP (all experiments)
L3R		TGTACCCATGAGCAAACTGC	ChIP (all experiments)
L400F	400	ATCTGACATGGCATTCCTCA	ChIP (all experiments)
L400R		GATGCCAGACCGTAATGACA	ChIP (all experiments)
L600F	600	CCATTGACTAGGAGGACTTTGAG	ChIP RPA
L600R		CCCTGGCGGTTGTAGTTAGT	ChIP RPA
L900F	900	AACGGTTGTAGAAGACGAGCA	ChIP RPA
L900R		TGTAAGCACACCTTCAATGTATCA	ChIP RPA
L1400F	1400	AACATCGGTGACCTCGTTCT	ChIP RPA
L1400R		CTCTTCGCTCCAAGCGTTAT	ChIP RPA
L2200F	2200	AAGGCAAGAAACGCTGAGAC	ChIP RPA
L2200R		GGCATGCATACTACCCGATAA	ChIP RPA
L3000F	3000	TCGTACGTACTGCATCGCTATAA	ChIP RPA
L3000R		TTCATCCTACCTTCTAGTTCGGTAA	ChIP RPA
II50F	Control locus on ChrII	CACCGCAGTTCTACGTATCCT	ChIP (all experiments)
II50R		CGATGTAACGGTATGCGGTA	ChIP (all experiments)
CenF	centromere (King et al 2008), cnt - center region	CAACCGTTGCAACTTACATCAGCA	ChIP Man1 and Sad1
CenR		CCGGTCGCCAAATAGCAATGAGAT	ChIP Man1 and Sad1
Lo5	ura4	GTTTAACTATGCTTCGTCGG	PCR screening (deletion/mutation)
Up5		AAAACAAACGCAAACAAGGC	PCR screening (deletion/mutation)
Rng3Lo	rng3	AAGGACTGCGTTCTTCTAGC	PCR screening efficiency control
Rng3Up		TGAATCCTCCGTTCAGTAGG	PCR screening efficiency control
TLII	TLII/TLIII	TTTCCTTTCACGGCTAACCC	PCR screening translocation
TLIII		TGTACCCATGAGCAAACTGC	PCR screening translocation
ade6-23	Control locus on ChrIII	GGCTGCCTCTACCATCATTC	ChIP (all experiments)
ade6-25		TTAAGCTGAGCTGCCAAGGT	ChIP (all experiments)
Ura4-1F	Within *ura4+* in proximity to lexBS; 600 bp away from RFB	GACTCCACGACCAACAATGA	ChIP lexA
Ura4-1R		CTGGTATCGGCTTGGATGTT	ChIP lexA
