# Supplementary material for: The nuclear pore primes recombination-dependent DNA synthesis at arrested forks by promoting SUMO removal
Source: Nat Commun. 2020 Nov 6;11:5643. doi: 10.1038/s41467-020-19516-z (PMC7648084; doi:10.1038/s41467-020-19516-z)
Supplement: Supplementary file 3 — Reporting Summary [file 41467_2020_19516_MOESM3_ESM.pdf]

nature research

Corresponding author(s): Sarah Lambert

Last updated by author(s): 2020/10/9

Reporting Summary

Nature Research wishes to improve the reproducibility of the work that we publish. This form provides structure for consistency and transparency in reporting. For further information on Nature Research policies, see Authors & Referees and the Editorial Policy Checklist .

Statistics

For all statistical analyses, confirm that the following items are present in the figure legend, table legend, main text, or Methods section.

n/a	Confirmed	
☐	☒	The exact sample size (n) for each experimental group/condition, given as a discrete number and unit of measurement
☐	☒	A statement on whether measurements were taken from distinct samples or whether the same sample was measured repeatedly
☐	☒	The statistical test(s) used AND whether they are one- or two-sided<br>*Only common tests should be described solely by name; describe more complex techniques in the Methods section.*
☒	☐	A description of all covariates tested
☒	☐	A description of any assumptions or corrections, such as tests of normality and adjustment for multiple comparisons
☐	☒	A full description of the statistical parameters including central tendency (e.g. means) or other basic estimates (e.g. regression coefficient) AND variation (e.g. standard deviation) or associated estimates of uncertainty (e.g. confidence intervals)
☐	☒	For null hypothesis testing, the test statistic (e.g. F, t, r) with confidence intervals, effect sizes, degrees of freedom and P value noted<br>*Give P values as exact values whenever suitable.*
☒	☐	For Bayesian analysis, information on the choice of priors and Markov chain Monte Carlo settings
☒	☐	For hierarchical and complex designs, identification of the appropriate level for tests and full reporting of outcomes
☒	☐	Estimates of effect sizes (e.g. Cohen's d, Pearson's r), indicating how they were calculated

Our web collection on statistics for biologists contains articles on many of the points above.

Software and code

Policy information about availability of computer code

Data collection	METAMORPH v 7.10.3.279 for cell miscrocopy aquisition (https://www.moleculardevices.com)<br>BIORAD CFX Maestro 1.1 for qPCR data collection<br>Typhoon trio (GE healthcare , software v 2.0.0.6) for collection of radiolabeled signal.
Data analysis	Fiji/Image J for cell microscopy analysis (https://imagej.nih.gov/ij/plugins/stack-contrast/index.htm)<br>Fiji/Image J plugin spotTracker (http://bigwww.epfl.ch/sage/soft/spottracker/)<br>MS Excel Macro for MSD analysis<br>MS Excel 2016 and GraphPadPrism 8 for analysis data management.<br>Image Quant v 5.1 (http://gelifesciences.com)

For manuscripts utilizing custom algorithms or software that are central to the research but not yet described in published literature, software must be made available to editors/reviewers. We strongly encourage code deposition in a community repository (e.g. GitHub). See the Nature Research guidelines for submitting code & software for further information.

Data

Policy information about availability of data

All manuscripts must include a data availability statement. This statement should provide the following information, where applicable:

- Accession codes, unique identifiers, or web links for publicly available datasets
- A list of figures that have associated raw data
- A description of any restrictions on data availability

Data have been deposited to Mendeley data and are available at: http://dx.doi.org/10.17632/4m7z3gy5yc.1
The source data underlying Figs 1d-e, 1h, 2b, 3b, 3d-g, 4b-c, 4e, 5c-e, 5g, 6b, 6e, 6g, 7a-c and Supplementary Figs 3b-c, 4c-d, 5b-c, 6c-d are provided as a Source Data file. All relevant data are available and further information and requests for reagents and resources should be directed to and will be fulfilled by Dr. Sarah A.E.

Lambert (sarah.lambert@curie.fr)

Spreadsheets for all the graphical representations are submitted as source data in excel files (.xls format)

Field-specific reporting

Please select the one below that is the best fit for your research. If you are not sure, read the appropriate sections before making your selection.

- [x] Life sciences
- [ ] Behavioural & social sciences
- [ ] Ecological, evolutionary & environmental sciences

For a reference copy of the document with all sections, see nature.com/documents/nr-reporting-summary-flat.pdf

Life sciences study design

All studies must disclose on these points even when the disclosure is negative.

Sample size	No statistical methods were used to predetermine sample size, as this study did not include animal models or human participants. Sample size was determined based on standards in the field and experiments to obtain statistical significance and reproducibility.<br>sample size for relocation at the nuclear periphery of at least 200 cells was chosen based on prior literature (Su et al. Gene and Dev 2015, ref 22).
Data exclusions	All data acquired for this study were included in the analysis
Replication	All experimental findings were reliably reproduced as indicated in the figure legends.
Randomization	No randomization was done beacuse this study did not involve animals or human participants. Samples were organized into groups based on treatment and genotype. Appropriate controls were included in all experiments.
Blinding	There was no blinded group allocation because of the nature of biological samples and type of experiments performed

Reporting for specific materials, systems and methods

We require information from authors about some types of materials, experimental systems and methods used in many studies. Here, indicate whether each material, system or method listed is relevant to your study. If you are not sure if a list item applies to your research, read the appropriate section before selecting a response.

Materials & experimental systems

n/a	Involved in the study	
☐	☒	Antibodies
☒	☐	Eukaryotic cell lines
☒	☐	Palaeontology
☒	☐	Animals and other organisms
☒	☐	Human research participants
☒	☐	Clinical data

Methods

n/a	Involved in the study	
☒	☐	ChIP-seq
☐	☒	Flow cytometry
☒	☐	MRI-based neuroimaging

Antibodies

Antibodies used	Antibody for ChIP were used as follows:<br>Anti-Rad51 (Abcam, ab63799) 1:300 dilution<br>Anti-GFP (invitrogen A11122) 1:150 dilution<br>Anti-LexA (Abcam, ab174384) 1:120 dilution<br>Rabbit IgG (Cell signaling technology, 2729s) 1:75
Validation	All antibodies were validated against strains that do not expressed relevant epitopes.<br>anti-Rad51: Telomerase Repairs Collapsed Replication Forks at Telomeres. Cell Rep. 2020 Mar 10;30(10):3312-3322.e3. doi: 10.1016/j.celrep.2020.02.065. PMID: 32160539.<br>Anti-GFP: Choi ES, Cheon Y, Kang K, Lee D. The Ino80 complex mediates epigenetic centromere propagation via active removal of histone H3. Nat Commun. 2017 Sep 13;8(1):529. doi: 10.1038/s41467-017-00704-3. PMID: 28904333; PMCID: PMC5597579.<br>anti-Lexa: Hishida T et al. Role of the Escherichia coli RecQ DNA helicase in SOS signaling and genome stabilization at stalled replication forks. Genes Dev 18:1886-97 (2004)<br>Rabbit IgG: Hu H, Ji Q, Song M, Ren J, Liu Z, Wang Z, Liu X, Yan K, Hu J, Jing Y, Wang S, Zhang W, Liu GH, Qu J. ZKSCAN3 counteracts cellular senescence by stabilizing heterochromatin. Nucleic Acids Res. 2020 Jun 19;48(11):6001-6018. doi: 10.1093/

nar/gkaa425. PMID: 32427330; PMCID: PMC7293006.

Flow Cytometry

Plots

Confirm that:

- [x] The axis labels state the marker and fluorochrome used (e.g. CD4-FITC).
- [x] The axis scales are clearly visible. Include numbers along axes only for bottom left plot of group (a 'group' is an analysis of identical markers).
- [x] All plots are contour plots with outliers or pseudocolor plots.
- [ ] A numerical value for number of cells or percentage (with statistics) is provided.

Methodology

Sample preparation	Cells fixed in 70 % ethanol were washed with 50 mM sodium citrate, digested with RNAse A (Sigma, R5503) for 2 hours, stained with 1µM Sytox Green nucleic acid stain (Invitrogen, S7020) and subjected to flow cytometry using FACSCANTO II (BD Biosciences).
Instrument	FACSCANTO II (BD Biosciences).
Software	FlowJo
Cell population abundance	10 000 events/sample
Gating strategy	Cell cycle analysis: Forward and side scatter parameters (FSC, SSC) were verified to exclude cell debris and doublets as shown in supplementary Figure 5. In general, 2-4% of events were excluded from analysis to remove noise signal at the boundaries of histogram of DNA content.

- [x] Tick this box to confirm that a figure exemplifying the gating strategy is provided in the Supplementary Information.
